# Exosomes and Other Extracellular Vesicles in HPV Transmission and Carcinogenesis

**DOI:** 10.3390/v9080211

**Published:** 2017-08-07

**Authors:** David Guenat, François Hermetet, Jean-Luc Prétet, Christiane Mougin

**Affiliations:** 1 EA3181, University Bourgogne Franche-Comté, LabEx LipSTIC ANR-11-LABX-0021, Rue Ambroise Paré, 25000 Besançon, France; david.guenat@univ-fcomte.fr (D.G.); jean_luc.pretet@univ-fcomte.fr (J.-L.P.); 2 CNR Papillomavirus, CHRU, Boulevard Alexandre Fleming, 25000 Besançon, France; 3 Department of Medicine, Division of Oncology, Stanford Cancer Institute, Stanford University, Stanford, CA 94305, USA; 4 INSERM LNC-UMR1231, University Bourgogne Franche-Comté, LabEx LipSTIC ANR-11-LABX-0021, Fondation de Coopération Scientifique Bourgogne Franche-Comté, 21000 Dijon, France; francois.hermetet@u-bourgogne.fr

**Keywords:** exosomes, microvesicles, apoptotic bodies, papillomavirus, horizontal gene transfer, cell-to-cell communication, carcinogenesis

## Abstract

Extracellular vesicles (EVs), including exosomes (Exos), microvesicles (MVs) and apoptotic bodies (ABs) are released in biofluids by virtually all living cells. Tumor-derived Exos and MVs are garnering increasing attention because of their ability to participate in cellular communication or transfer of bioactive molecules (mRNAs, microRNAs, DNA and proteins) between neighboring cancerous or normal cells, and to contribute to human cancer progression. Malignant traits can also be transferred from apoptotic cancer cells to phagocytizing cells, either professional or non-professional. In this review, we focus on Exos and ABs and their relationship with human papillomavirus (HPV)-associated tumor development. The potential implication of EVs as theranostic biomarkers is also addressed.

## 1. Introduction

Extracellular vesicles (EVs) including exosomes (Exos), microvesicles (MVs), and apoptotic bodies (ABs) have recently attracted great attention in cancer research. EVs take part in cell-to-cell communication between cancer cells and neighboring cells, including fibroblasts and endothelial and immune cells [1]. A growing body of evidence indicates that EVs can modulate tumor progression. Their pro-tumorigenic properties are fully described and reviewed elsewhere [2,3,4,5,6,7]. These properties are linked to direct effects through horizontal transfer of bioactive molecules (mRNAs, microRNAs (miRNAs), DNA or proteins) in recipient cells, and to indirect effects by remodeling the cancer microenvironment.

One of the most exciting applications of EV research in cancer is their potential use as biomarkers to monitor cancer progression by means of liquid biopsy, a non-invasive procedure [8]. In recent years, an exponential number of methods for minimally invasive early detection, diagnosis and follow-up of cancers has been developed using either circulating tumor cells (CTCs) or circulating tumor DNA (ctDNA). Circulating EVs from liquid biopsy may advantageously be used instead of CTCs or ctDNA. Indeed, they bear crucial protein and genetic material in their cargo and studying them may potentially provide information on the cellular origin of cancer. Circulating EVs are released virtually by all tumor cells as well as by the tumor microenvironment. Thus, the study of circulating EVs gives a snapshot of the heterogeneity of both the tumor and the microenvironment. The current limitation of liquid biopsy is the fragmentation of circulating DNA and the poor conservation of circulating RNA. Nucleic acids associated with EVs seem to be well conserved, perhaps shielded from degradation by nucleases inside the lipid bilayer membranes of the vesicles. This offers an opportunity for minimally invasive whole genome and/or whole transcriptome analysis from the blood of any cancer patient. Sequencing of both exosomal DNA and RNA may detect all driver and passenger mutations, translocations and amplifications which can be molecular targets for new drugs [3,9].

Few studies related to the role of EVs in virus transmission or carcinogenesis are available in the literature. Published reports describe how EVs modulate viral cell life, activate proliferation pathways, or modify the microenvironment to promote viral-associated carcinogenesis [10].

Although the role of EVs derived from human papillomavirus (HPV)-induced cancer cells is not yet completely understood, we review here recent data demonstrating their importance in tumorigenesis.

## 2. Extracellular Vesicles and Cancer

### 2.1. Biogenesis and Characterization of Extracellular Vesicles

**Figure 1:**
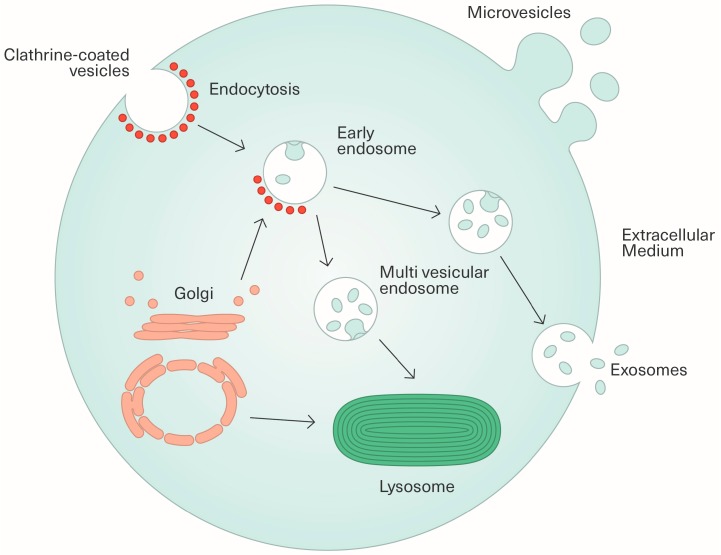
Exosome and microvesicle biogenesis. Exosomes originate from the inward budding of endosomes to form multivesicular endosomes (MVEs). MVEs then fuse with the cell surface membrane to release exosomes into the extracellular medium. Some MVEs fuse with lysosomes. Microvesicles are produced by direct budding from the plasma membrane.

“Extracellular vesicle” is a term given to a heterogeneous group of particles released by cells. A range of different names, referring to several types of EVs, can be found in the literature, such as exosomes, ectosomes, oncosomes, prostasomes, apoptotic bodies, shedding vesicles, microparticles or microvesicles amongst others, but no consensus on their classification has emerged thus far. However, based on the mechanisms of biogenesis, the scientific community classically distinguishes three main subgroups of EVs, namely exosomes, microvesicles and apoptotic bodies. Exos were first described in 1983 by Harding et al. [11]. These small vesicles, initially considered as a way to eliminate altered and/or unnecessary cellular constituents, originate from the invagination of endosome-forming multivesicular endosomes (MVEs). MVEs then fuse with the cell surface membrane to liberate Exos in the extracellular compartment. MVs refer to a subgroup of EVs produced by direct budding at the cell membrane (Figure 1). ABs are vesicles shed by the fragmentation of cells following the induction of apoptosis. The diameter of vesicles precludes specifically distinguishing different types of EVs from each other, but it is usually accepted that the size of Exos ranges mostly from 30 to 100 nm (approximately overlapping the size of virus particles), the size of MVs from 100 nm to 1 µm, and the size of ABs from 1 to 5 µm [12].

EVs derive from a large proportion of normal and cancer cell lines in vitro. In vivo, EVs can be isolated in most (if not all) biofluids, including plasma, urine, cerebrospinal fluid, saliva or breast milk and are produced in physiological and pathological conditions, especially by cancer cells. EV isolation protocols based on differential ultracentrifugation are probably the gold standard [13,14] and allow for the recovery of high amounts of EVs with intact physicochemical and functional properties. Highly pure EV preparations can be obtained with an additional step of density gradient ultracentrifugation [15]. Nevertheless, these protocols are time-consuming. In order to reduce the laboratory turnaround time, alternative EV isolation methods including commercial kits based on immunocapture, chromatography, microfluidics or polyethylene glycol precipitation have been developed by different research groups and companies in a very competitive market [16,17,18,19].

To date, international guidelines regarding the standardization of pre-analytical methods (specimen handling, isolation protocols) and analytical techniques to characterize EVs (electron microscopy, nanoparticle tracking analysis, proteomics) are not clearly defined and would be useful to choose the most relevant procedure for specific applications [20]. This is why the International Society for Extracellular Vesicles published recommendations defining the minimal requirements (e.g., biochemical, biophysical and functional standards of EVs) to conduct research on EVs [21]. Recently, a worldwide survey confirmed the great diversity of methods used by scientists working on EVs; it further pointed out that the EV field is rapidly evolving and that the standardization of practices remains challenging [22].

Of note, given the absence of specific markers of Exos or MVs, it remains difficult to clearly distinguish the origins of EVs after isolation. Thus, most of the published works on Exos probably relies on mixes of Exos and MVs and it should be kept in mind that the term “exosomes” generally refers to EVs or exosomes-enriched EVs. In the different sections below we will use the proper term EVs, except when referring to articles in which the authors used the term “Exos” instead of EVs.

### 2.2. Content and Functions of Extracellular Vesicles

EVs are packed with proteins, lipids and nucleic acids that mirror the parental cell-of-origin [23]. Studies have shown that Exos are specifically enriched in some biologically active molecules via a non-random process whose molecular mechanism remains unknown [24]. In particular, cytoskeletal proteins (actin, cofilin), adhesion molecules (tetraspanins, integrins), peptide-binding proteins such as the heat shock protein family proteins, major histocompatibility complex (MHC) class I and II, and membrane fusion proteins (*Ras* genes from rat brain (RAB) family proteins, annexins) are enriched in Exos and may serve as biomarkers. Interestingly, the exosomal nucleic acid content, mainly mRNA and miRNA, can constitute a specific molecular signature to characterize Exos. Some authors have reported that Exos contain high molecular weight double-stranded DNA. Using comparative genomic hybridization array [25] or, more recently, whole genome sequencing [26], it has been shown that exosomal DNA represents the entire genome of the parental cells. Moreover, in cancer patients, the exosomal DNA is enriched with tumor DNA [26]. From a structural point of view, one unsolved question concerns the association of nucleic acids with Exos. Indeed, to the best of our knowledge, no study has clearly demonstrated whether exosomal nucleic acids are located within vesicles, adsorbed onto the vesicle surface, or simply co-purified with vesicles as nucleic acid–protein complexes [12]. The methods used to isolate Exos are likely to influence their nucleic acids cargo. Thus, the physiological and pathological functions of nucleic acids associated with Exos remain a matter of debate.

ABs represent the ultimate stage of apoptosis. Indeed, apoptosis is presented as a three-stage process: an initiation phase followed by an integration phase and finally an irreversible execution phase (reviewed in [27]). Specific ultrastructural, biochemical and mechanical modifications lead to AB formation [28,29]. Caspases activated by apoptotic signals constitute the main agents of cellular demolition through the cleavage of numerous cytoplasmic (e.g., constituents of the cell cytoskeleton) and nuclear (e.g., nuclear envelope) substrates. From a dynamic point of view, apoptotic cells undergo plasma membrane blebbing followed by karyorrhexis and formation of ABs which are eliminated by phagocytes. ABs present characteristic morphological features. They are delineated by a plasma membrane whose phosphatidylserines are located at the outer leaflet. The plasma membrane of ABs also exhibits intrinsic proteins, some of which serve as “eat me” signals recognized by receptors expressed on phagocytes. Furthermore, ABs contain altered mitochondria, fragmented organelles (Golgi apparatus, endoplasmic reticulum) and fragments of nucleus with typical internucleosomal DNA fragmentation [28,29].

**Figure 2:**
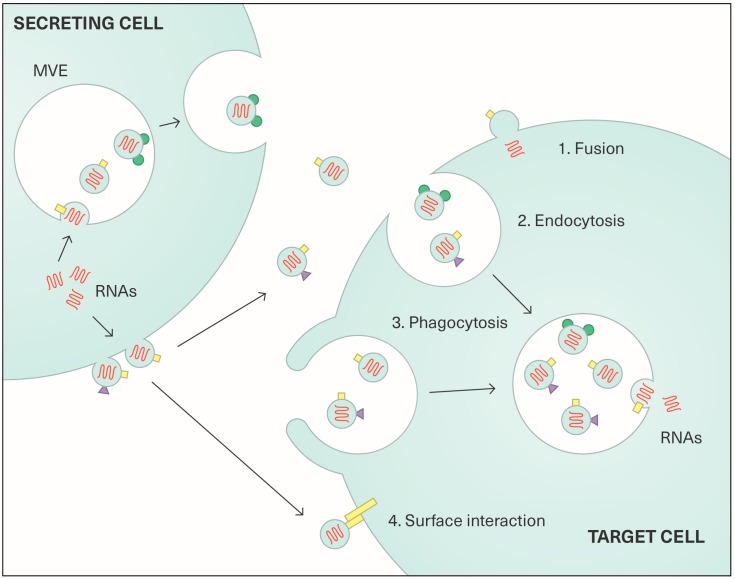
The four different modes of communication by exosomes and microvesicles. Extracellular vesicles serve as vehicles for cell-to-cell communication through horizontal transfer of bioactive molecules (proteins, lipids and nucleic acids). Extracellular vesicles (microvesicles (MVs) or exosomes (Exos)) produced from a secreting cell may be internalized by fusion (1), endocytosis (2), phagocytosis (3) or may interact with the membrane proteins of the target cell (4). Squares, triangles and circles represent membrane-associated proteins.

EVs may be considered as vehicles for local and systemic cell-to-cell communication. Indeed, EVs are small messengers that interact with target cells, directly via cell surface molecules, or after endocytosis and internalization of bioactive molecules (Figure 2).

The function of EVs has long been studied, especially by immunologists because of their important role in antigen presentation. For instance, EVs have the potential to present antigens via MHC molecules and to activate cellular immune responses. However, efficient priming of naïve T cells seems to be restricted to Exos released from mature dendritic cells and requires the expression of intercellular adhesion molecule 1 [30]. Exos can also activate the host innate immunity. Indeed, Exos derived from cells infected by intracellular pathogens such as mycobacteria [31] or directly derived from pathogens such as *Leishmania* [32] can spread molecular patterns of infection leading to the modulation of the host immune responses.

Cell-to-cell communication also involves the transfer of biologically active molecules to target cells following the internalization of Exos. Valadi et al. showed that mRNA purified from mast cell Exos were functional as they could be translated in vitro [33]. More interestingly, when exosomal mRNAs isolated from mouse cells were delivered to human cells, the corresponding mouse proteins were efficiently expressed and detected in the recipient cells. Thus, it appears consistent that Exos are readily able to reprogram the phenotype of recipient cells via the transfer of RNA [12]. Nevertheless, numerous questions regarding processing, delivery and functions of EV-associated RNA remain unsolved and important considerations have to be properly addressed to refine understanding of RNA components [34].

Horizontal transfer, also called lateral transfer, of malignant traits from one mammalian cell to another involves cancer pathogenic effectors like DNA, mRNA, miRNA, lipids, proteins, transcription factors and cell-surface receptors circulating as free molecules or via tumor-derived MVs or Exos [35,36,37,38,39]. It can also occur through the clearance of apoptotic cancer cells, termed efferocytosis [40,41,42,43]. During cancer progression, many cells are lost through apoptosis [44]. Growth/survival factor depletion, hypoxia, loss of cell-matrix interactions and DNA damage are implicated in cell death [45]. Thereafter, professional phagocytic cells largely contribute to dead cell clearance by inactivating and degrading cellular components [46]. Over the last few decades, it has become clear that amateur phagocytes like epithelial cells [47,48,49] or fibroblasts [48,50] are able to engulf apoptotic corpses. Through this endocytic process, apoptotic cells can act as a vector of biomolecules which may confer a selective advantage on the recipient cell.

### 2.3. Pro-Tumorigenic Properties of Exosomes

Cancer-derived Exos can activate transforming signaling pathways in recipients cells by transferring oncogenic proteins such as mutated Kirsten rat sarcoma viral oncogene homolog (KRAS) proteins [51] or the epidermal growth factor receptor variant III [52]. This generally leads to enhanced growth of the target cells, sometimes in an anchorage-independent manner, and in this regard Exos participate in the horizontal transfer of oncogenes. Exos also have the ability to remodel the tumor microenvironment by fostering angiogenesis. Umezu et al. confirmed that multiple myeloma-derived Exos promote the angiogenic potential of endothelial cells. This occurs via the transfer of miR-135b that targets factor-inhibiting hypoxia-inducible factor 1 (HIF-1). Consequently, this releases HIF-1 which in turn can execute its transcriptional activity to maintain oxygen homeostasis [53]. Tumor-derived Exos were also shown to favor the expression of numerous angiogenic factors such as vascular endothelial growth factor (VEGF), VEGF receptor 2 or von Willebrand factor following their engulfment by endothelial cells. Furthermore, they are important mediators that induce the differentiation of fibroblasts into a “wound-healing” myofibroblastic phenotype that is associated with tumor growth [54]. Recently, Peinado et al. reported that Exos have a major role in the dissemination of tumor cells at distant sites. In particular, they showed that tumor-derived Exos educate bone marrow cells to prepare a pre-metastatic niche that will favor the recruitment and growth of metastasizing cancer cells [55,56]. Last but not least, cancer-derived Exos can modulate anti-tumor immune responses. Since they express tumor-specific antigens at their surface, Exos derived from cancer cells can elicit anti-tumor immune responses. Specifically they can transfer tumor antigens to dendritic cells which in turn induce efficient CD8+ T-cell-dependent antitumor effects [57]. Based on these observations, the concept of exosome-based anti-tumor vaccines has been proposed, but the translation of this into the clinic is tempered by the fact that cancer-derived Exos mostly present immunosuppressive effects. Indeed, cancer-derived EVs mediated both direct and indirect immunosuppressive effects notably through the modulation of inflammatory mediators. First, they harbor immunosuppressive molecules such as Fas-Ligand or tumor-necrosis-factor related apoptosis inducing ligand (TRAIL), check point receptor ligands (PD-L1) or inhibitory cytokines (interleukin-10, transforming growth factor-β1, and prostaglandin E2) [6]. They can induce the cell death of CD95+ lymphocytes [58], impair the activity of natural killer cells, and decrease the proliferative response of CD8+ T cells to interleukin-2 due to the proliferation of forkhead box P3 (Foxp-3) regulatory T cells [59]. Second EVs promote the proliferation of myeloid-derived suppressor cells, favor the polarization of macrophages toward a M2 phenotype, or inhibit the differentiation of lymphoid or myeloid progenitors (for review see [60]). Finally, tumor antigens at the surface of Exos may represent decoy targets for antibodies, thereby impairing antibody-dependent cell-mediated cytotoxicity [61] or limiting the bioavailability of therapeutic antibodies such as trastuzumab [62].

### 2.4. Horizontal Oncogene Transfer by Apoptotic Bodies

In 1999, de la Taille et al. revealed for the first time that tumor cells derived from a prostate adenocarcinoma (LNCaP) are able to exchange genetic information [63]. LNCaP^bcl−2/neo−r^ cells highly resistant to in vitro apoptotic signals were cocultured with LNCaP^hygr−r^ cells sensitive to serum starvation apoptosis. Cocultures gave rise to progeny cells containing both genetic markers when they were exposed to an apoptotic stimulus that preferentially kills LNCaP^hygr−r^ cells. This provides evidence that genetic information can be transferred from apoptotic cells to viable partners through the phagocytosis process. The authors termed this latter event as an apoptotic conversion and put forward the hypothesis that this process has the potential to mediate the passage of genetic defects between cancer cells in a tumor, leading to the development of more aggressive cancer cell types after therapy of some cancer patients. In the same year, Holmgren et al. demonstrated that irradiation-induced ABs of Burkitt-derived lymphocytes harboring integrated Epstein-Barr virus (EBV) DNA can transfer viral sequences to professional and non-professional phagocytes such as endothelial cells and fibroblasts during co-cultivation [64]. Recipient human macrophages and bovine aortic endothelial cells expressed viral sequences (Epstein-Barr nuclear antigen 1, Epstein-Barr virus-encoded RNA 1 and 2) at a higher frequency than human fetal fibroblasts, which is likely related to their higher phagocytic activity. Interestingly, viral DNA fragments are preferentially transferred compared to human DNA. However, horizontal EBV DNA transfer was not observed when lymphocyte cell lines harboring episomal copies of EBV were cocultured with recipient cells. This suggests that viral episomes were more prone to degradation by apoptosis-activated nucleases than integrated DNA copies.

Two years later, Holmgren’s team reported that DNA could be horizontally transferred from apoptotic c-myc and H-ras^V12^-transfected rat embryonic fibroblasts (REF) to p53^−/−^ mouse embryonic fibroblasts (MEF) resulting in loss of contact inhibition and anchorage-independence in vitro as well as a tumorigenic phenotype in severe combined immunodeficiency (SCID) mice [65]. When ABs derived from normal REF were exposed to recipient cells, none of the above effects was observed. Similarly, ABs were not capable of conferring the transformed characteristics of cancer cells onto recipient cells with intact p53. Thus, in phagocytic recipient cells, genomic instability results from the horizontal transfer of malignant traits and the inactivation of the *tp53* tumor suppressor gene [65]. Afterwards, Bergsmedh et al. showed that MEF cells lacking the p21^Cip1/Waf1^ cyclin-kinase inhibitor are able to propagate DNA after the uptake of ABs derived from a rat fibrosarcoma, resulting in foci formation in vitro and tumor growth in SCID mice [66]. Taken together, the data from Holmgren’s team indicate that p53 via the activation of its target p21 protects recipient cells from the propagation of potentially pathological exogenous DNA acquired from apoptotic cells [66]. This protective mechanism is dependent on DNA fragmentation and *DNase II* together with the *Chk2* DNA damage pathway, to form a protective barrier against horizontally transferred DNA [67].

More recently, Ehnfors et al. demonstrated that DNA from apoptotic rat fibrosarcoma cells expressing H-ras^V12^, c-myc and simian virus SV40 large T (SV40LT) can be transferred to, and transform fibroblasts and endothelial cells in vitro [68]. Interestingly, tumor DNA can be spontaneously transferred to stromal murine host cells in vivo. A sub-population of endothelial cells expressing SV40LT and injected together with Matrigel into SCID mice form new functional blood vessels. Inactivation of the p53 pathway through the transfer of SV40LT might overcome the barrier to tumor DNA propagation [68].

## 3. Exosomes in Viral Transmission and Carcinogenesis Associated with Viruses Other than HPV

Studies agreed that EVs may enhance viral transmission and carcinogenesis in different models and this has been recently reviewed [69]. Ramakrishnaiah et al. showed that Exos derived from hepatitis C virus (HCV)-infected cells contain full-length viral RNA. Furthermore, these Exos were capable of delivering viral RNA into non-infected cells and establishing a productive infection [70]. This mode of transmission, which does not depend on the production of infectious virions, may be an efficient mechanism for the virus to escape the immune system since Exos derived from HCV-infected cells are not neutralized by immunoglobulin (Ig) Gs purified from HCV-infected patients.

Since Exos are constitutively produced by cells, it may be considered that their composition is altered following viral infection. To address this question, Meckes et al. investigated the protein composition of Exos derived from uninfected B cells or Epstein–Barr virus (EBV) or Kaposi sarcoma-associated herpesvirus (KSHV)-infected B cells [71]. Two hundred and thirty proteins were specifically expressed in virus-infected cells, 93 of them being specific to EBV-infected Exos and 22 to KSHV-infected Exos. In EBV-infected Exos, the main oncogenic viral protein latent membrane protein 1 (LMP1) was systematically detected, confirming a previous study conducted from a lymphoblastoid cell line infected by EBV [72]. LMP1-enriched Exos also exhibit high levels of other proteins that are associated with cancer and supposed to affect in particular survival/proliferation/death pathways, cell movement, cell-to-cell signaling as well as protein synthesis pathways and immune response [71,73,74,75]. Interestingly, Exos derived from both EBV and KSHV-infected cell lines also harbor the Ezrin protein, which is absent from the parental non-infected B cell line. This protein, a plasma membrane-microfilament linker involved in cellular architecture, was shown to activate the protein kinase B survival pathway in epithelial cells [76].

The involvement of LMP1-enriched Exos (also called LMP1^+^Exos) in oncogenesis can be considered in several ways. First, LMP1^+^Exos can remodel the tumor microenvironment by facilitating the secretion of the angiogenic fibroblast growth factor 2 [77]. Second, it has been shown, at least in vitro, that LMP1^+^Exos are readily engulfed through the endocytic pathway [75] leading to the activation of several intracellular pathways in recipient cells. For example, the engulfment of LMP1^+^Exos by B cells activates their proliferation and their differentiation in plasmablast-like cells [74]. As for nasopharyngeal carcinoma, EBV-derived Exos are readily able to induce an epithelial–mesenchymal transition to recipient cells. They also favor the metastatic potential of the recipient cells through HIF-1 α [73]. Moreover, EBV-modified Exos can play a role in immune evasion by inducing T cell anergy and protecting neighboring tumor cells from immune effector cells [72,78]. Furthermore, galectin-9-containing Exos derived from nasopharyngeal cancer cells induce the apoptosis of EBV-specific CD4+ T cells. The binding of galectin-9 to the death-inducing receptor anti-T-cell immunoglobulin and mucin-domain containing-3 (Tim-3) expressed by mature T helper (Th)1 cells appeared necessary given that anti-Tim-3 and antigalectin-9-blocking antibodies tempered the suppressive activity of Exos [79].

## 4. Extracellular Vesicles and HPV-Associated Carcinogenesis

Persistent infection with high risk (hr) human papillomaviruses (HPV) is the necessary cause of cervical cancer [80]. Among hrHPV, HPV16 and HPV18 are responsible for more than 70% of invasive cervical cancers in the world [81]. Expression of the viral E6 and E7 proteins is crucial for cellular immortalization, transformation and maintenance of the transformed phenotype [82]. E6 and E7 oncoproteins target many cellular growth regulatory circuits, among them the guardian of the genome protein p53 [83] and the growth-suppressive form of the retinoblastoma (Rb) tumor suppressor Rb Protein (pRb) [84], respectively. Interestingly, silencing of E6/E7 in HeLa cervical carcinoma cells reactivates tumor suppressor pathways leading to cellular senescence [85,86].

### 4.1. E6/E7 and Anti-Apoptotic Exosomal Proteins

It has been previously shown that various cancer cell lines, among which HeLa, an HPV18-infected cell line derived from cervix adenocarcinoma, actively release Exos harboring survivin [87,88], a member of the inhibitory of apoptosis protein (IAP) family. Other members of the IAP protein family (cellular inhibitor of apoptosis protein (c-IAP), X-linked inhibitor of apoptosis protein1/2 (XIAP1/2)) were also identified in exosomal fractions released from HeLa cells, but only mRNA encoding survivin was detected [89]. In a model of HeLa cells stably transfected to overexpress survivin, it has been shown that a sublethal dose of proton irradiation significantly increases the survivin content of Exos, but not the secretory rate of Exos [88].

Since tumor virus infection modulates the composition of Exos, Honegger et al. determined whether E6/E7 could alter the amounts and/or contents of Exos released from HPV-infected cancer cells [90,91]. To address this question, the authors used a small interfering RNA (siRNA) approach to knock-down E6/E7, bearing in mind that a genome-wide transcriptome analysis previously showed that survivin mRNA was downregulated following E6/E7 repression [92]. In the experiments of Honegger et al., E6/E7 expression was effectively downregulated in HeLa cells by siRNA for up to 96 h and the authors confirmed that survivin expression was reduced. As for Exos, no major modification in their morphology was noted following E6/E7 downregulation but their number was largely increased when compared to mock-treated cells. Regarding the content of IAP family members, the relative level of survivin was lower in exosomal fractions derived from E6/E7 siRNA treated cells than from control cells. Nevertheless, the relative level of survivin remained higher in exosomal fractions than in whole cell extracts, indicating that survivin is specifically enriched in Exos, even if E6/E7 is downregulated. The authors also documented the presence of XIAP, c-IAP1 and livin alpha in Exos, raising the question of whether these vesicles could play a role in modulation of apoptosis.

In HPV-associated cancer models, it has been shown that conditioned media obtained from cells transfected with survivin construct present anti-apoptotic properties and protect HeLa cells from ultra-violet (UV)-induced apoptosis [87]. Thus, survivin liberated in the microenvironment via Exos may act as a protumorigenic factor [87,88,89,90,91]. Indeed, these Exos are assumed to be engulfed by neighboring cancers cells that in turn become more proliferative and resistant to therapy.

### 4.2. E6/E7 and miRNA Content of Exosomes

Honegger et al. investigated the exosomal miRNA content using a small RNA deep sequencing approach and compared it to the miRNA pattern expressed from Exo-producing HeLa cells whose E6/E7 expression was silenced [91]. They first observed an absence of 18S and 28S RNA in exosomal fractions confirming previous publications, but clearly identified transfer RNAs and miRNAs. Then, they documented that the relative levels of miRNAs among small RNAs were 50% less abundant in Exos than in exosome-producing cells. While E6/E7 silencing did not affect the intracellular content of miRNA in whole cells, it increased two-fold the relative miRNA content in Exos, suggesting that E6/E7 regulate exosomal miRNA sorting. Among the 47 most expressed exosomal miRNAs, 21 were upregulated and 4 were downregulated following E6/E7 silencing. The modulation of miRNA expression following E6/E7 siRNA treatment was further confirmed by real-time quantitative PCR (RT-qPCR) for approximately three-quarters of the targets and among them, let-7d-5p, miR-20a-5p, miR-378a-3p, miR-423-3p, miR-7-5p, miR-92a-3p were downregulated and miR-21-5p was upregulated. Interestingly, this signature of seven miRNAs was altered in a similar manner in Exos derived from E6/E7 siRNA-treated SiHa cells, a HPV16-infected cell line derived from cervix squamous cell carcinoma, suggesting that exosomal miRNA content is neither dependent on the type of tumor tissue nor on HPV genotype, but most likely on E6/E7 expression.

Very recently, Harden and Münger assessed whether HPV16 E6/E7 expression altered miR expression in Exos derived from human foreskin keratinocytes (HFKs). After E6/E7 retroviral transduction, both intracellular and Exo miRNA contents were examined and compared to non-transduced HFKs [93]. Forty-eight miRNAs were deregulated in E6/E7 HFKs and 31 were differentially expressed in Exos showing that E6/E7 oncoproteins alter miRNA composition of Exos. HPV16 E6/E7 expression modifies the expression of many miRNAs in a similar manner intracellularly and in Exos. However, seven miRNAs were either upregulated or downregulated in Exos when compared to whole cells: miR-16-5p, miR-21-5p, miR-200b-3p, miR-205-5p, miR-222-3p, miR-320a, miR-378-3p. Interestingly, the biological functions of miRNAs selectively packaged in Exos are predicted to inhibit apoptosis and necrosis [93]. These results are consistent with those published by Honegger et al. in their E6/E7 extinction model [91]. They also confirm the data published by Chiantore et al. who demonstrated that specific miRNAs were packaged in Exos derived from HFKs transduced with HPV16 E6/E7 [94].

Thus, tumor-promoting activity of Exos derived from hrHPV-positive cells might rely on the transfer of oncogenic miRNAs to recipient cells. Indeed, most of the miRNAs altered by E6/E7 display oncogenic activities. Among these miRNAs, miR-222 appears to be of particular interest since it was shown to play a role in cervical carcinogenesis, notably through the downregulation of p27 and phosphatase and tensin homolog deleted on chromosome 10 (PTEN) [95]. Moreover, the miR-7-5p favors cell proliferation [96], the miR-20a-5p blocks oncogene-induced senescence by targeting p21 [97], and miR-92a-3p possesses anti-apoptotic properties [98].

### 4.3. Exosome Release and Senescence Induction

E6/E7 siRNA-treated HeLa cells release higher amounts of Exos than control cells and become senescent but not apoptotic. Interestingly, the relationship between Exo release and senescence has also been reported for irradiated prostate cancer cells [99]. This secretory phenotype was clearly dependent on the reactivation of p53, which in turn activates the transcription of the tumor suppressor-activated pathway 6 (*TSAP6*) and the charge multivesicular body protein 4C (*CHMP4C*) encoding proteins involved in Exo production/sorting [100,101,102]. Honegger et al. investigated *TSAP6* and *CHMP4C* transcript levels in E6/E7 siRNA-treated HeLa cells [90]. Silencing endogenous HPV18 E6/E7 oncogene expression led to reconstitution of p53 linked to increased expression of *TSAP6* and *CHMP4C* contributing to senescence induction. While such a senescence should reduce tumor growth, it favors the release of a broad spectrum of molecules associated with inflammation and malignancy [103]. This is probably why senescence is associated with cancer progression in certain circumstances [104], and secretion of IAP family protein-enriched Exos (especially when E6/E7 are targeted in HPV-associated cancer) may participate in tumor development/progression.

### 4.4. Exosomes as Potential Biomarkers

Numerous studies have reported biological effects mediated by EVs. However, we should keep in mind that experimental procedures do not reflect physiological conditions. Therefore, the in vitro observations have to be cautiously interpreted. Nevertheless, given that Exos released from HPV-associated cancer cells present key features, they may serve as very specific cancer biomarkers [105]. For this purpose, analysis of Exos purified from cervico-vaginal lavage samples of patients with cervical cancer made it possible to demonstrate high levels of miR-21 and miR-146a [106] or long non-coding RNAs (especially metastasis associated lung adenocarcinoma transcript 1 (MALAT1) which is known to be activated by E6) [107]. Thus, like liquid biopsies taken from blood sampling [108,109], analysis of Exos from cervico-vaginal lavages may serve as a non-invasive procedure for early diagnosis and follow-up of patients with HPV-associated cancers.

### 4.5. HPV-Positive Apoptotic Bodies and Cell Transformation

In our laboratory, we investigated whether HPV16 and HPV18 might be capable of conferring transformed characteristics of apoptotic cancer cells onto normal recipient human fibroblasts, and we hypothesized that the horizontal transfer of HPV oncogenes could be an alternative mechanism of carcinogenesis [40]. We provided evidence that apoptotic wild-type p53 HeLa cells (harboring integrated HPV18) and Ca Ski cells (derived from a metastatic cervical epidermoid carcinoma harboring integrated HPV16) but not p53-mutated and HPV-negative C-33 A cells (derived from a rare HPV-negative cervical carcinoma) were able to transform human primary fibroblasts (HPFs) [40,42]. HPFs engulf late apoptotic cells more efficiently than early apoptotic cells, but their phagocytic activity remains limited compared to professional phagocytes [41,43]. The uptake of ABs requires phosphatidylserine recognition, which is mainly mediated by brain-specific angiogenesis inhibitor 1 (BAI-1). Microtubule-associated protein1-light chain 3 (LC3)-associated phagocytosis (LAP) is required for the clearance of dying cells, as demonstrated by confocal microscopy analysis with organelle-specific markers [41,43] and is well described in the review of Green et al. [110]. To the best of our knowledge, this is the first time that LAP has been observed in amateur phagocytes. However, its significance in the clearance of apoptotic corpses and possible altered intracellular processing, especially in the context of horizontal gene transfer (HGT), remains to be clarified. While there is accumulating data for a role of cancer-associated fibroblasts in cancer progression [111,112], studies are necessary to clarify the role of efferocytosis during HPV-induced tumor development, metastasis and chemotherapy-mediated tumor clearance.

HPFs with phagocytosed apoptotic HeLa and Ca Ski cells were able to form colonies when grown under anchorage-independent conditions [40,41,42], which is a major hallmark of cellular transformation [113]. This process was further tested by limit-dilution assays. Horizontal transfer of viral oncogenes in fibroblast recipients was confirmed using in situ hybridization with a probe hybridizing specifically with hrHPV DNA [40,42]. Moreover, the expression of E6 transcripts studied by RT-qPCR were detected in the transformed recipient cells with, however, lower levels than in the parental HeLa and Ca Ski cells. In addition, the levels of p53 and p21 in the host cells decreased substantially. Chromosomal rearrangements (loss and gain of alleles) were confirmed by studying microsatellite amplifications. As described by Holmgren, small and large DNA fragments were transferred [64]. Chromosomal instability and aneuploidy observed in our model are common characteristics of cancer cells that increase the acquisition of mutations conferring aggressive or drug-resistant phenotypes during cancer progression [114,115]. Our findings suggest that horizontal transfer of viral oncogenes may confer the transformed characteristics of cancer cells on fibroblasts and epithelial cells, which are major constituents of the tumor microenvironment [40,41,42,43]. Even though we did not highlight the whole complexity of cell transformation following engulfment of ABs, the inactivation of p53 through the transfer of HPV oncogenes likely disrupts the protective barrier against tumor development.

In 2012, Trejo-Becerril et al. [116] hypothesized that circulating oncogenic DNA, also called virtosome [117], spontaneously released by living cells, was able to foster tumor progression. Indeed, the authors confirmed that cancer progression is mediated by HGT when animals were injected in the dorsum with human SW480 colon carcinoma cells as a source of circulating oncogenic DNA. However, results were less meaningful when immunocompetent rats were intraperitoneally injected daily for 30 days with supernatant of HeLa cells or when intravenously injected every day for 60 days with ABs from HeLa cells, which were obtained after exposure to 75 µM cisplatin for 24 h. In this last condition, no clinical alteration was observed and no dysplastic or neoplastic cells were found in different organs. To provide evidence of nucleic acid transfer, PCR and RT-PCR targeting E6 and E7 of HPV18 were performed. Viral DNA was detected in the liver, spleen, colon, uterus, lung and kidney while viral mRNAs were expressed only in the liver. Ongoing research in our laboratory shows that apoptotic HeLa cells subcutaneously injected in the flank of Swiss nu/nu mice led to tumor formation at the injection site. Major organs (kidney, liver, lung, spleen) are currently under investigation.

Our findings shed important light on unconventional and poorly understood mechanisms of cell-to-cell communication and how that communication may have significant consequences in human HPV-associated cancer progression. Transfer of oncogenic HPV DNA sequences from cancer epithelial cells to fibroblasts makes it possible to explain the detection of HPV DNA in stromal cells of unusual undifferentiated cervix carcinomas [118].

## 5. Conclusions

There is increasing evidence in support of the pro-tumorigenic properties of Exos derived from cancer cells. These properties rely either on a direct effect of Exos by transferring oncogenes to recipient cells or on an indirect effect by remodeling the microenvironment. In virus-infected cancer cells, it is noteworthy that Exos composition is modified compared to non-infected cells. These EVs appear to be enriched with viral or cellular oncogonenic factors, such as LMP1, survivin or certain oncomiRs. Thus, viral-modified Exos might be involved in intercellular communication by delivering oncogenic molecules to the neighboring cells. On the other hand, they are armed to manipulate the tumor environment by favoring angiogenesis or exerting immunosuppressive effects, for example. ABs can also serve as efficient vectors for carrying oncogenic signals to normal cells. Indeed, they can transfer viral and cellular oncogenes to amateur phagocytes cells through HGT and induce their immortalization/transformation. Further investigations are necessary to better understand these alternative modes of oncogenesis mediated by EVs. Together, this will lead to an improved understanding of viral carcinogenesis and will offer new opportunities in cancer diagnostics and therapeutics [119].
